# Digital Magnetic Sorting for Fractionating Cell Populations with Variable Antigen Expression in Cell Therapy Process Development

**DOI:** 10.3390/magnetochemistry10110081

**Published:** 2024-10-23

**Authors:** Savannah Bshara-Corson, Andrew Burwell, Timothy Tiemann, Coleman Murray

**Affiliations:** Ferrologix, Inc., Valencia, CA 91354, USA

**Keywords:** cell therapy manufacturing, digital magnetic sorting, antigen density

## Abstract

Cellular therapies exhibit immense potential in treating complex diseases with sustained responses. The manufacture of cell therapies involves the purification and engineering of specific cells from a donor or patient to achieve a therapeutic response upon injection. Magnetic cell sorting targeting the presence or absence of surface markers is commonly used for upfront purification. However, emerging research shows that optimal therapeutic phenotypes are characterized not only by the presence or absence of specific antigens but also by antigen density. Unfortunately, current cell purification tools like magnetic or fluorescence-activated cell sorting (FACS) lack the resolution to differentiate populations based on antigen density while maintaining scalability. Utilizing a technique known as digital magnetic sorting (DMS), we demonstrate proof of concept for a scalable, magnetic-based approach to fractionate cell populations based on antigen density level. Targeting CD4 on human leukocytes, DMS demonstrated fractionation into CD4^Hi^ T cells and CD4^Low^ monocytes and neutrophils as quantified by flow cytometry and single-cell RNA seq. DMS also demonstrated high throughput processing at throughputs 3–10× faster than FACS. We believe DMS can be leveraged and scaled to enable antigen density-based sorting in cell therapy manufacturing, leading to the production of more potent and sustainable cellular therapies.

## 1. Introduction

Adoptive cell therapies taking advantage of engineered chimeric antigen receptors (CARs) or T cell receptors (TCRs) have shown incredible potential as “living drugs” that achieve personalized immunotherapies for cancer patients [1]. As the field expands beyond liquid tumors and into other disease indications, the need to develop therapies based on more well-defined and specialized cell populations is growing [2,3]. Typically, manufacturing cellular therapies requires the enrichment of specific cell types from a donor or patient before genetic engineering and expansion. In many cases, a specific phenotype drives the therapeutic response. For example, the CD34+ CD90+ stem cell subpopulation shows superior engraftment compared to single-positive CD34 hematopoietic stem cells [4]. However, a cell population’s functional performance is not only characterized by the presence or absence of markers but also by subsets defined by surface marker density. For example, CD4 is highly expressed on certain T cell populations (~98 K antigens/cell) but is also expressed at low levels on some monocytes and neutrophils (~50–20 K antigens/cell) [5–7]. Additionally, natural killer cell populations can be differentiated into CD56^Hi^ and CD56^Low^, which correlate to cytokine activity or higher cytotoxicity phenotypes [8]. For engineered CART cells, having sufficient CART antigen density is a critical metric of therapeutic performance, where too little antigen density leads to insufficient anti-tumor activity and too much can lead to tonic signaling and off-target effects [9,10]. The ability to fractionate cell populations by both antigen presence and density would be a significant capability for cell therapy production and expand the field’s capabilities. However, current cell isolation methods cannot achieve the scale and cost effectiveness required for cell therapy manufacturing while also having sufficient resolution to differentiate variable antigen density. Magnetic-activated cell sorting (MACS) is a standard technique in cell therapy manufacturing, but its binary nature prevents it from reliably differentiating high vs. low antigen populations. Fluorescence-activated cell sorting (FACS) has sufficient resolution to differentiate high-vs. low-expressing populations but lacks the capability to scale to manufacturing levels because it is an intrinsically serial process where one cell is measured at a time and requires expensive antibody multiplexing. Some microfluidic technologies are in development for variable antigen isolation but also face scalability challenges, are susceptible to clogging, and are too complex or expensive to manufacture under cGMP [11–15].

Digital magnetic sorting (DMS), also known as ratcheting cytometry, utilizes single-use cartridges containing arrays of ferromagnetic microstructures that can isolate different populations of cells based on the level of magnetic content bound to the surface of each cell [16–21] while operating in a parallel fashion like standard MACS. By generating a cycling magnetic field, provided by the DMS benchtop instrument, cells can be captured and fractionated into discrete populations based on the magnetic intensity of each cell, similar to how FACS sorts based on light intensity.

In this work, we evaluated the ability for DMS and a superparamagnetic bead cocktail to fractionate CD4+ cells into CD4^Hi^ and CD4^Low^ populations corresponding to T cells, monocytes, and neutrophils. It has been well documented that CD4 is highly expressed on CD4 T helper cells and expressed at lower levels on monocytes and some neutrophils [5–7]. Leveraging DMS’ multiplexed sorting capabilities, we enriched CD4^Hi^ cells into a High Magnetic Fraction (HMF), which could be differentiated from CD4^Low^ cells in the Low Magnetic Fraction (LMF) within the DMS cartridge. The capability for DMS to fractionate cells based on antigen expression at throughputs conducive to cell therapy process development and manufacturing (from 10^8^ to 10^9^ target cells/h) and can potentially expand the capabilities of cell therapy manufacturing to generate therapeutic cells based on specific antigen-level cell populations and improve therapeutic efficacy or even procure optimized subpopulations of engineered cells based on a threshold of a specific marker.

## 2. Materials and Methods

### 2.1. Biological Samples

Human buffy coat samples were procured from San Diego Blood Bank (CAT # WBBC-1F). Ficoll gradient separation (Ficoll-Paque+, Cytivia) was performed using standard protocol to procure all leukocytes. Leukocyte samples were then cryopreserved in aliquots of 1 × 10^7^ cells/vial and stored in a −80 °C freezer until used.

### 2.2. Magnetic Labeling CD4^Hi^ CD4^Low^ Populations

Leukocyte samples were thawed and resuspended in working buffer (PBS–calcium containing 0.5% BSA + 0.1 mM EDTA) to a volume of 500 μL. Moreover, 50 μL of Ferrologix proprietary CD4 magnetic bead cocktail (SKU: DMS-0-CD4) was added to samples ranging from 1 × 10^7^ to 4 × 10^7^ cells. The samples were placed on a rotor at 18 RPM in the fridge (4 °C) for 30 min.

### 2.3. DMS of High and Low Populations

After magnetic labeling, all magnetic cells were selected using a quadrupole magnet separator (Biomagnetic Solutions QP-5) to select all CD4+ cells (a process we call “preselection”). Cells were allowed to magnetically precipitate for 5 min and washed with 2 mL of working buffer. The cells were then removed from the magnetic separator and resuspended in 1 mL of working buffer. The sample was then loaded onto a Ferrologix DMS cartridge (SKU: DMS-0-CRT) primed with working buffer and separated under the “Dynamic Load” program at a magnetic separation frequency of 20 Hz for a 30 min duration. After DMS processing, the cartridge extraction lock valve was actuated to separate the HMF from LMF. The cells were then eluted from the DMS cartridge via extraction ports [21].

### 2.4. Flow Cytometry, Cell Counting, and Viability

**Figure A1.**
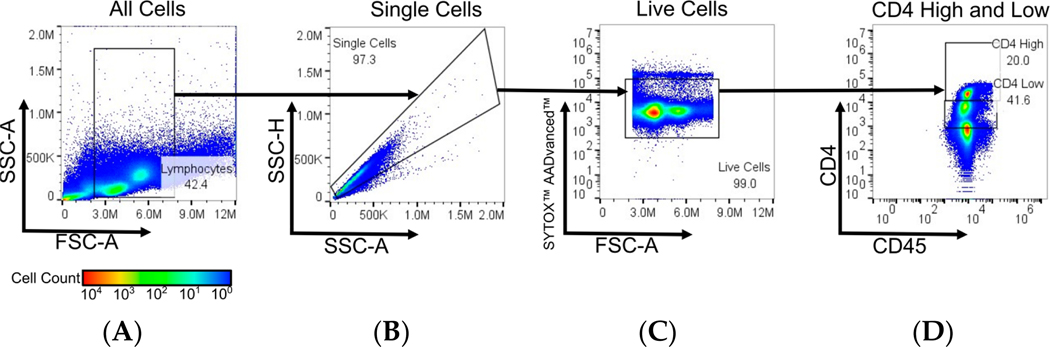
(**A**) Initial gating on lymphocytes was performed on the native sample, utilizing forward scatter (FSC) and side scatter (SSC) parameters to segregate the lymphocyte population based on their intrinsic physical properties. (**B**) The ‘Single Cells’ gate was established to ensure analysis of individual cell events, thereby excluding multi-cell aggregates and enhancing the accuracy of the subsequent gating steps. (**C**) Viability assessment was conducted using Thermo Fisher Scientific’s SYTOX^™^ Advanced^™^ Dead Cell Stain Kit (Catalog No. S10349). This step involved gating live cells to exclude non-viable cells, ensuring the analysis focused solely on live cellular constituents. (**D**) Discrimination of CD4^Hi^ and CD4^Low^ cell populations was then performed. CD4^Hi^ cells, typically representative of T cell populations, were gated based on their characteristic expression patterns. In contrast, CD4^Low^ cells, correlating with expected monocyte populations, were gated accordingly. This distinction was pivotal in reflecting the anticipated frequencies of these cell types within peripheral blood mononuclear cells (PBMCs).

Antibodies that were used were from Biolegend. FITC anti-human CD14 antibody (CAT # 367116), PE anti-human CD4 antibody (CAT # 317410), and APC anti-human CD45 antibody (CAT #304012). SYTOX^™^ Advanced^™^ Dead Cell Stain Kit (CAT #S10274) was acquired from Thermo Fisher Scientific to determine the viability of cells post-DMS. Cells were counted using immunofluorescence microscopy using a Keyence microscope. Each field of view is assessed using two optical channels: the bright field channel, which displays an image of the captured cells, and the viability channel, which employs a fluorescently labeled cell viability stain (Calcein AM). Calcein AM green was used to determine how many cells were in each sample for each step of the protocol. Flow cytometry data were analyzed with FlowJo v10.5 per the gating strategy in Appendix A, Figure A1. See the Supplementary Material folder “Flow Cytometry Data” for FCS files.

### 2.5. Single-Cell RNA Seq of High and Low Populations

Cells were processed into HMFs and LMFs and titrated to a concentration of 1 × 10^5^ cells/μL. The cells were then added into a Chromium^™^ Next GEM Chip (10× Genomics) and processed through a Chromium Controller for generation of single-cell RNA seq libraries. Following the manufacturer’s protocol, cDNA libraries were generated and sequenced on an Illumina Sequencer. FASTQ files were aligned and analyzed via the 10× Cloud Analysis System. Loupe Browser files were analyzed using standard quality control methods with Q30 scores >90%. Cell categorization was performed using the 10× recommended gene list for leukocytes. Differential CD4 expressions between LMF and HMF were also performed for different cell categories (See the Supplementary Material folder “scRNA Seq Data”).

## 3. Results

### 3.1. DMS Workflow

**Figure A2.**
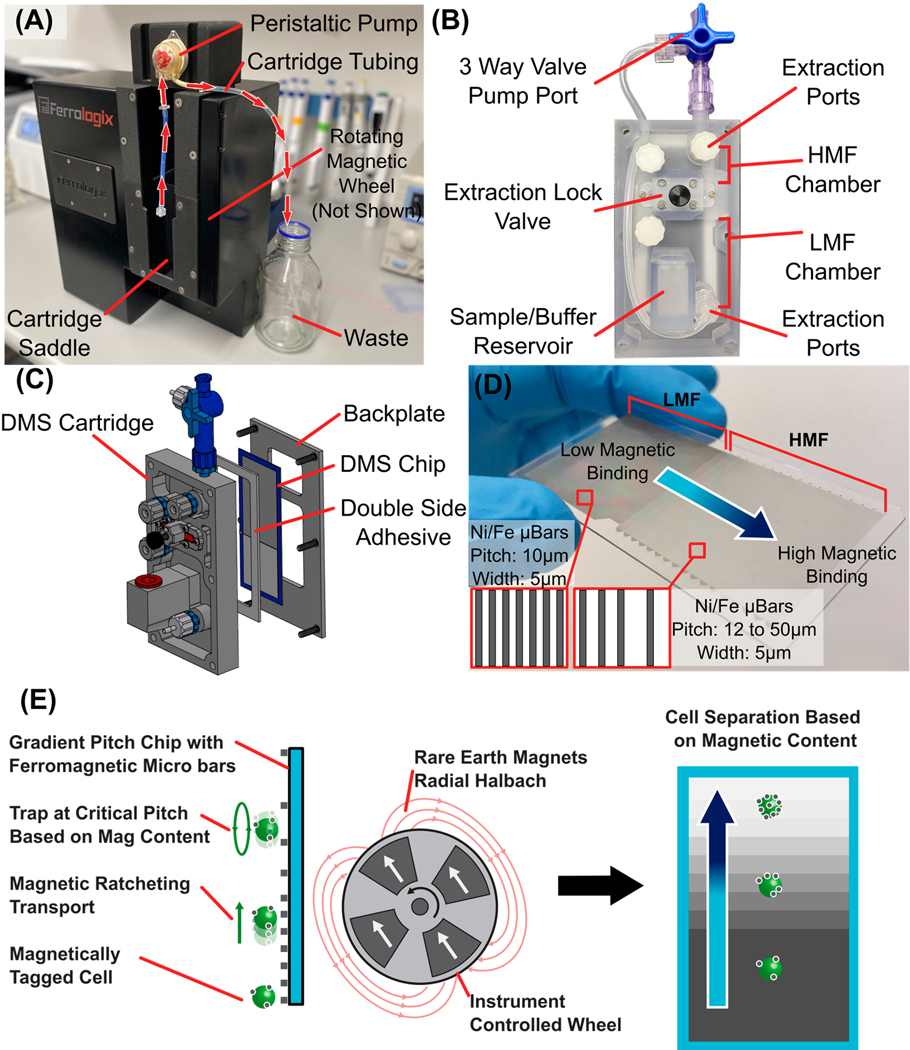
Primer on digital magnetic sorting (DMS), also known as ratcheting cytometry. (**A**) The DMS benchtop instrument accepts a DMS cartridge into a “saddle”, which is then hooked to a tubing and peristaltic pump to draw fluid through the cartridge. Internal and centered with the cartridge is a rotating magnetic wheel in radial Halbach array orientation. This rotating wheel drives the digital magnetic sorting behavior across the magnetic chip within the cartridge. (**B**) The cartridge itself consists of a buffer/sample input reservoir, a flexible extraction lock valve to sequester LMF and HMF regions in the chip, a three-way Luer Lock valve to connect to the peristaltic pump, and Luer Lock extraction ports to remove cells. (**C**) The DMS cartridge is a clamshell assembly containing the DMS magnetic chip, which is secured to the cartridge chamber via a backplate and die-cut double-sided adhesive strip. (**D**) The DMS chip itself is a 25 × 75 mm glass substrate with regions of high-density nickel/iron micro-bars with a 5 μm width, 25 mm length, and a thickness between 3 and 5 μm. The first region is the LMF and is 25 × 25 mm with a microbar pitch of 10 μm. This region is directly connected into the HMF region, which consists of multiple 2 mm long × 25 mm wide sections of increasing pitch. The first region has a 12 μm pitch and then increments by 2 μm with each 2 mm section (12 μm, 14 μm, 16 μm, . . ., 50 μm). (**E**) In response to a rotating magnetic field generated by the DMS benchtop instrument, the microbars magnetize in alignment to the bulk field, modifying the magnetic potential energy landscape and introducing potential wells in which superparamagnetic particles/cells migrate. As the wheel is cycled, particles/cells will follow the potential energy wells and jump from bar to bar against gravity based on size and bound iron oxide content. Using a chip consisting of microstructure arrays with gradient horizontal pitch, cells with different levels of magnetic binding will traverse the array until reaching their critical pitch, where they will collect and oscillate. Magnetized cells with increasing magnetic content will have correspondingly higher critical pitches and can therefore be separated. This results in temporally stable distributions of cells based on levels of magnetic content per cell or particles per cell. In this way, cell populations can be differentiated based on their level of magnetic binding (Denoted by vertical gradient arrow). Another analogy to conceptualize the process of digital magnetic sorting is to imagine the cells are climbing a “magnetic ladder” whose rungs are moving further apart the higher they climb. Magnetized cells will “jump” to a higher rung (or Ni/Fe microbar) with each cycle of the magnetic wheel as they chase the dynamic potential energy wells up the chip. At some point the magnetically tagged cells will have insufficient strength to jump to the next rung of the ladder. This “critical pitch” is determined by the amount of magnetic content bound to the cell, the pitch of the Ni/Fe microbars, and the characteristics of the magnetic field at that point on the chip (flux density [T] and rotational frequency of the wheel). This enables highly magnetic cells to climb the chip further than weakly magnetic cells, enabling multi-population sorting based on bound magnetic content.

Digital magnetic sorting (also known as ratcheting cytometry) is a novel technology that can quantitatively sort magnetically labeled cells based on their surface-level expression correlating to the number of beads present on a cell [18–20]. Magnetically tagged cells travel across a chip composed of ferromagnetic microstructures that are subjected to a directionally cycled magnetic field. The DMS benchtop system contains a rotating magnetic wheel composed of rare earth magnets, which drive magnetic particles across the ferromagnetic structure to enrich and transport cells when an instantaneous field is applied. In this way, the microstructure array generates an oscillating microscale field, which causes magnetized cells to continuously transport across the chip/cartridge. By introducing a gradient in the microstructure pitch (i.e., the distance between ferromagnetic elements), target cells will separate and trap at increasing distant spatial locations across the chip contingent upon increasing amounts of magnetic content on its surface (Figure A2). Depending on the number of particles bound to the cell, the cells will snap to the corresponding location or specific critical pitch at a given field frequency [19]. Leveraging DMS’ quantitative magnetic sorting capabilities, cells can be targeted with superparamagnetic particles such that cells will become magnetized in proportion to their antigen density, i.e., higher antigen density cells will become more magnetized than lower antigen density cells and can be differentiated using DMS [20].

**Figure 1.**
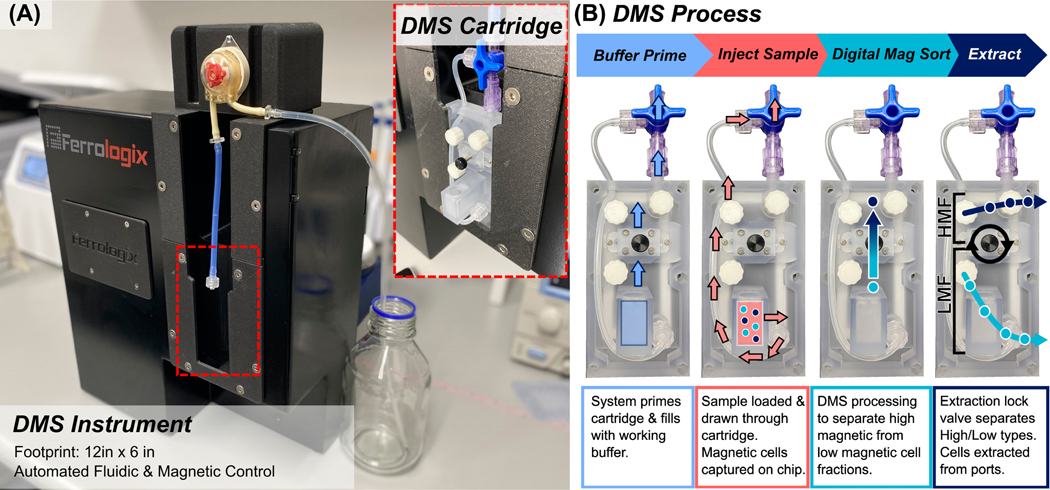
(**A**) The DMS instrument houses a closed loop controlled internal magnetic wheel of rare earth magnets arranged in a Halbach array orientation to generate a cycling magnetic field and a peristaltic pump to perform automated sample processing through the DMS cartridge. The DMS cartridge is loaded into the benchtop DMS instrument and connected to the peristaltic pump. A tablet with a simple graphic user interface walks the user through a standardized protocol. (**B**) The DMS cartridge workflow consists of: (1) Buffer priming to fill the cartridge with working buffer. (2) Sample injection, where the magnetically tagged cell sample is circulated through the base of the cartridge chamber, where magnetized cells are captured and pulled onto the micromagnetic substrate. (3) Digital magnetic sorting, where cells are transported vertically across the micromagnetic substrate and separate into High Magnetic and Low Magnetic Fractions. (4) Extraction after DMS separation. A flexible “extraction lock” valve is actuated to separate the High Magnetic Fraction from the Low Magnetic Fraction. Each fraction can be eluted from the cartridge via Luer Lock ports.

The DMS technology is automated through a benchtop instrument with a single-use cartridge format, as shown in Figure 1. The benchtop instrument contains an automated wheel of rare earth magnets (Figure A2) to drive magnetic separations as well as an on-board peristaltic pump to perform automated fluid handling operations and is controlled via a simple graphic user interface on a tablet computer. The DMS cartridge consists of a plastic clamshell assembly that encloses a magnetic gradient microstructure chip to form a sterile cartridge (see Figure A2).

**Figure A3.**
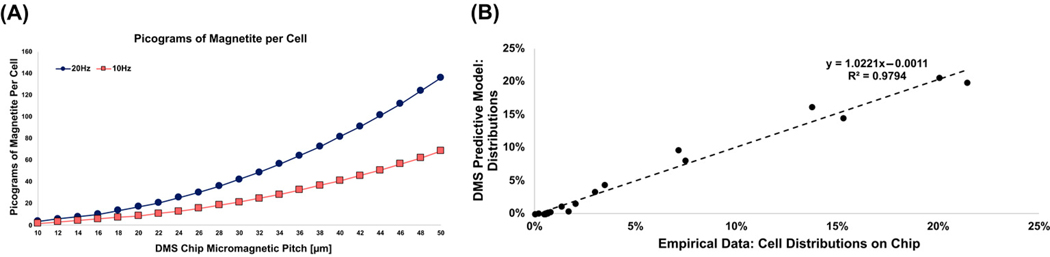
Numerical modeling of magnetic content per cell. We have already demonstrated in previous work [19] that cells transport and concentrate onto magnetic pitch ranges depending on the microelement pitch, cell-bound magnetic content, and the magnetic field frequency. (**A**) Using the numerical model developed previously, we can determine what level of iron oxide content per cell corresponds to a specific magnetic microstructure pitch under a given magnetic frequency. (**B**) The predictive model matches well with multiple empirical datasets where individual cells were fluorescently imaged through DMS chips to quantify the number of bound magnetic beads per cell with known levels of magnetic content per bead.

**Figure A4.**
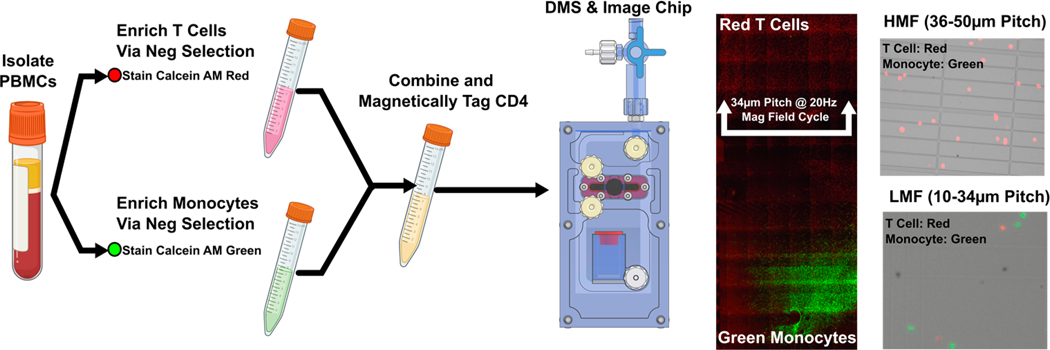
T cell and monocyte distributions on the DMS chip. Two key system and cartridge design parameters needed to be considered for fractionating the high and low antigen density populations: Primarily the frequency of the magnetic field that the magnetic cartridge was subjected to and the micromagnetic pitch range that the cells magnetically equilibrate to. From numerical modeling, we know the ranges that cells separate to with varying magnetic contents per cell. Empirical separations needed to be carried out to correlate CD4 surface expression on human cell populations with iron oxide content and ultimately, micropillar pitch ranges and frequencies on the DMS cartridge. To achieve this, we procured pre-fractionated PBMC samples, which were split into T cells and monocytes (custom order from AllCells, Inc., Alameda, CA, USA). Using established assays from previous work [20], we stained the T cells with calcein AM red and the monocytes with calcein AM green to create a two-population mixture. After magnetic labeling and separation on a DMS chip, we fluorescently imaged the chip (a) to determine where the populations equilibrated under various magnetic labeling conditions and magnetic frequencies. (b) Image analysis enabled us to develop distributions of T cells vs. monocytes and determine that a 20 Hz separation frequency and a cutoff off pitch of 34 μm enabled maximum separability between the CD4^Hi^ T cells and CD4^Low^ monocytes/neutrophils. T cells (red) separated above the 34 μm pitch at a 20 Hz frequency, while the monocytes were mainly retained in the bottom, below the 34 μm pitch. This experiment set allowed us to optimize DMS separation parameters and cartridge designs to determine physical cartridge cutoff locations (extraction lock location and separation frequency).

Samples of magnetized cells of up to 5 mL can be loaded into a Luer Lock reservoir and processed through the cartridge, where target cells are first pulled onto the magnetic chip loading zone and separated vertically into extraction regions. Actuatable valves, known as extraction locks, can be dropped to sequester different cell fractions corresponding to on-chip locations and extracted via Luer Lock ports (Figure 1B). Specifically, this DMS cartridge fractionates magnetized cells into two fractions: High Magnetic Fraction (HMF) and Low Magnetic Fraction (HMF). The LMF corresponds to magnetic microelement pitch ranges between 10 μm and 34 μm, while the HMF corresponds to microelement pitch ranges between 36 μm and 50 μm. Under a 20 Hz magnetic frequency, the LMF corresponds to an iron oxide content per cell of 3.5 to 56 picograms per cell, and the HMF corresponds to an iron oxide content per cell of 64 to 135 picograms under a 20 Hz separation (Appendix A, Figure A3). In this way, DMS can fractionate populations based on high vs. low magnetic content, which correlates with antigen density (Figure A4).

### 3.2. DMS Fractionation of CD4^Hi^ and CD4^Low^ Cell Types

**Figure 2.**
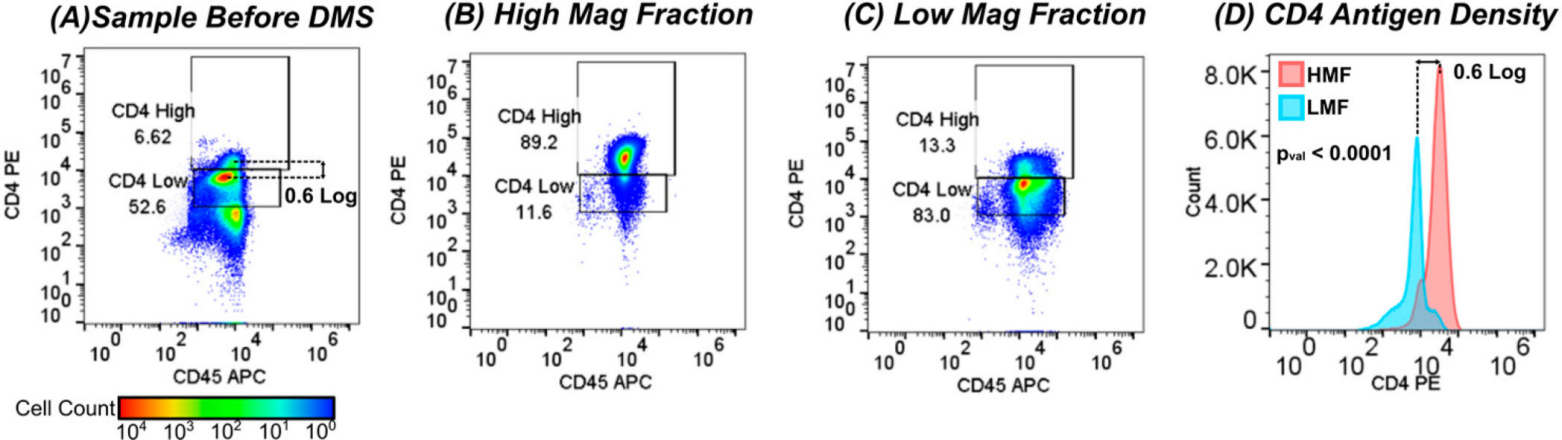
Flow cytometry analysis of leukocytes processed through the DMS high/low workflow. (**A**) Before separation, the sample was observed to have CD4^Hi^ and CD4^Low^ populations, with >99% of the cells being CD45+. The peak-to-peak difference between CD4^Hi^ and CD4^Low^ populations was observed to be approximately 0.6 log. (**B**) The HMF demonstrated enrichment of CD4^Hi^ cells with 81.8% ± 4.1 (N = 3 trials) of cells occupying the CD4^Hi^ gate, while (**C**) the LMF showed enrichment of CD4^Low^ cells with 78.8% ± 4.3% (N = 3 trials). (**D**) Histogram plots of CD4 expression between HMF and LMF show significant peak expression with a peak-to-peak difference that correlates with the original sample. ANOVA analysis of the HMFs and LMFs showed statistically significant differences in CD4 expression (*p* value < 0.0001).

Figure 2 shows the flow cytometry outputs before separation and after separation into High Magnetic Fraction (HMF) targeting CD4^Hi^ and Low Magnetic Fraction (LMF) targeting CD4^Low^. Initially, the population consisted of CD45+ 6.62% CD4^Hi^ T cells and 52.6% CD45+ CD4^Low^ monocytes. Note that the CD4^Hi^ and CD4^Low^ populations can be readily observed in the sample corresponding to the T cell and monocyte populations (Figure 2A). In this way flow cytometry gates were established to delineate the High/Low Magnetic Fractions. The leukocyte sample was then magnetically tagged using a proprietary superparamagnetic bead cocktail targeting CD4. Following magnetic tagging, CD4+ cells (both high and low) were concentrated using a standard quadrupole tube magnet to enrich all labeled cells. After enrichment of all CD4+ cells, the sample was processed through the DMS workflow as shown in Figure 1B.

**Figure 3.**
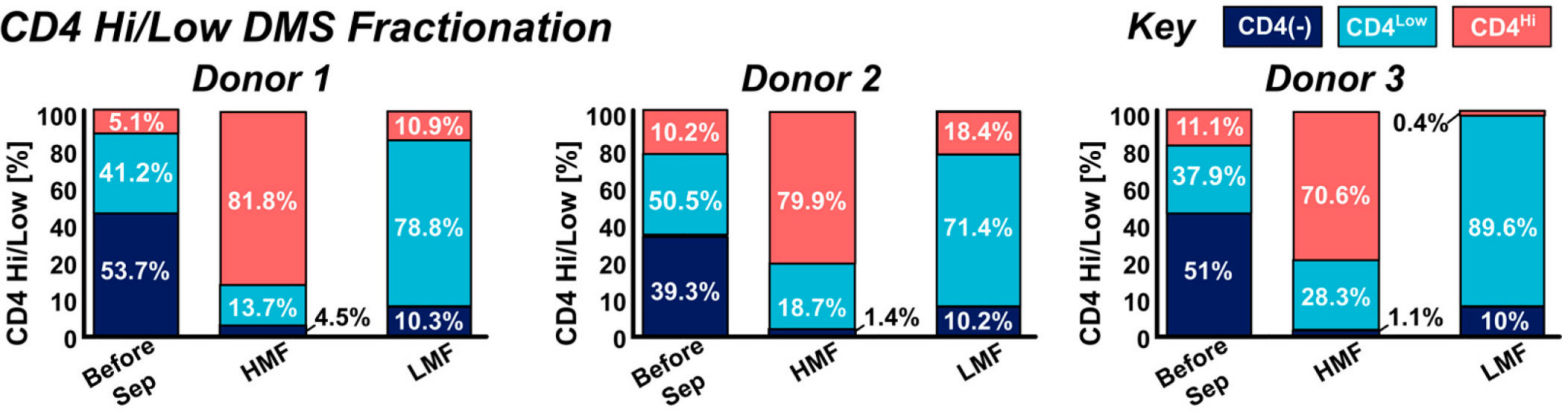
Multi-donor fractionation performance of the DMS system of CD4^Hi^ and CD4^Low^ populations into the High Magnetic Fraction (HMF) and Low Magnetic Fraction (LMF), respectively. Donor samples had varying ratios of CD4(−), CD4^Hi^, and CD4^Low^ phenotypes typically split between 40 and 50% CD4(−), between 40 and 50% CD4^Low^, and from 5 to 10% CD4^Hi^. Phenotypic gating based on CD4 expression was carried out using flow cytometry of samples before separation and in the LMFs and HMFs. Separations across multiple donors show high enrichment of CD4^Hi^ cells in the HMF (77.4 ± 4.9% donor average) and high enrichment of CD4^Low^ cells in the LMF (79.9 ± 7.5% donor average). All DMS fractionations show >90% purity of CD4(+) cells in both HMFs and LMFs.

**Figure A5.**
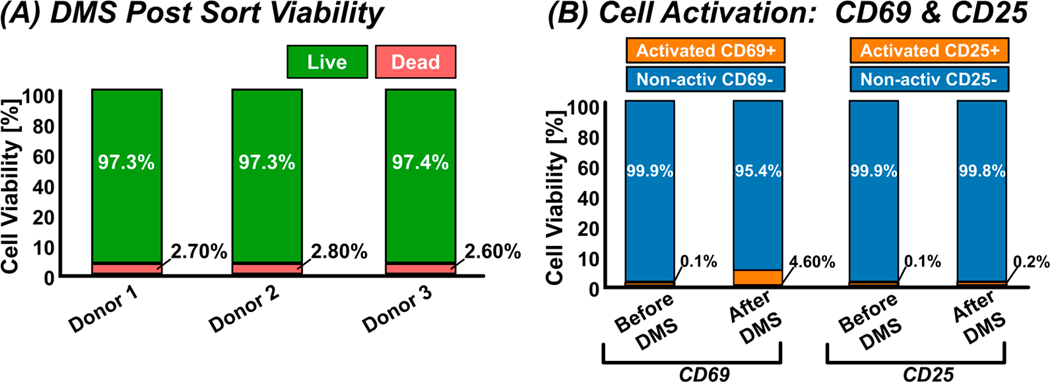
Quantification of cell viability and activation from digital magnetic sorting. (**A**) Cell viability after DMS sorting was ascertained via flow cytometry (SYTOX^™^ Advanced^™^ Dead Cell Stain Kit, Thermo Fisher Scientific, Carlsbad, CA, USA) and was found to be 97.3 ± 0.05% across three independent donors. (**B**) Analysis of cell activation on sorted T cells was also performed using flow 1 h after DMS processing, observing CD69 (early activation) and CD25 (late activation). We observed the population of CD69+ cells being slightly elevated (from 0.1% to 4.6%) compared to CD25, which showed no observable increase in CD25+ T cells. Some early-stage activation of cells is expected in any cell processing step but still shows a minor impact on cell activation.

After elution from the cartridge, the cells were quantified via flow cytometry. The HMF showed high enrichment of the CD45+ CD4^Hi^ T cell fraction of 81.8 ± 4.1% of total cells within the CD4^Hi^ gate (Figure 2B). The LMF consisted of both CD4+ T cells and CD4+ monocytes but had significantly lower CD4 expression, where 78.8 ± 4.3% of cells fell within the CD4^Low^ gate (Figure 2C). Histogram plots of CD4 expression between the HMF and LMF show statistically significant differences in CD4 expression as quantified by ANOVA differential analysis (*p* value < 0.0001) and over a 0.6 log difference in peak-to-peak expression, which correlates with the log difference in the unsorted sample. These data show that the DMS system is capable of rapidly fractionating populations based on antigen density with high viability and minimal cell activation (Figure A5) in a single step and exhibits significant advantages over traditional, binary magnetic sorting methods. In total, three donors were processed in triplicate with DMS high/low sorting to fractionate different antigen density populations. Figure 3 shows the summarized results of the percentage of cells within each CD4^Hi^/CD4^Low^ flow cytometry gate within the HMF and LMF extractions.

### 3.3. Single-Cell RNA Seq Confirms Antigen Density Separation

**Figure 4.**
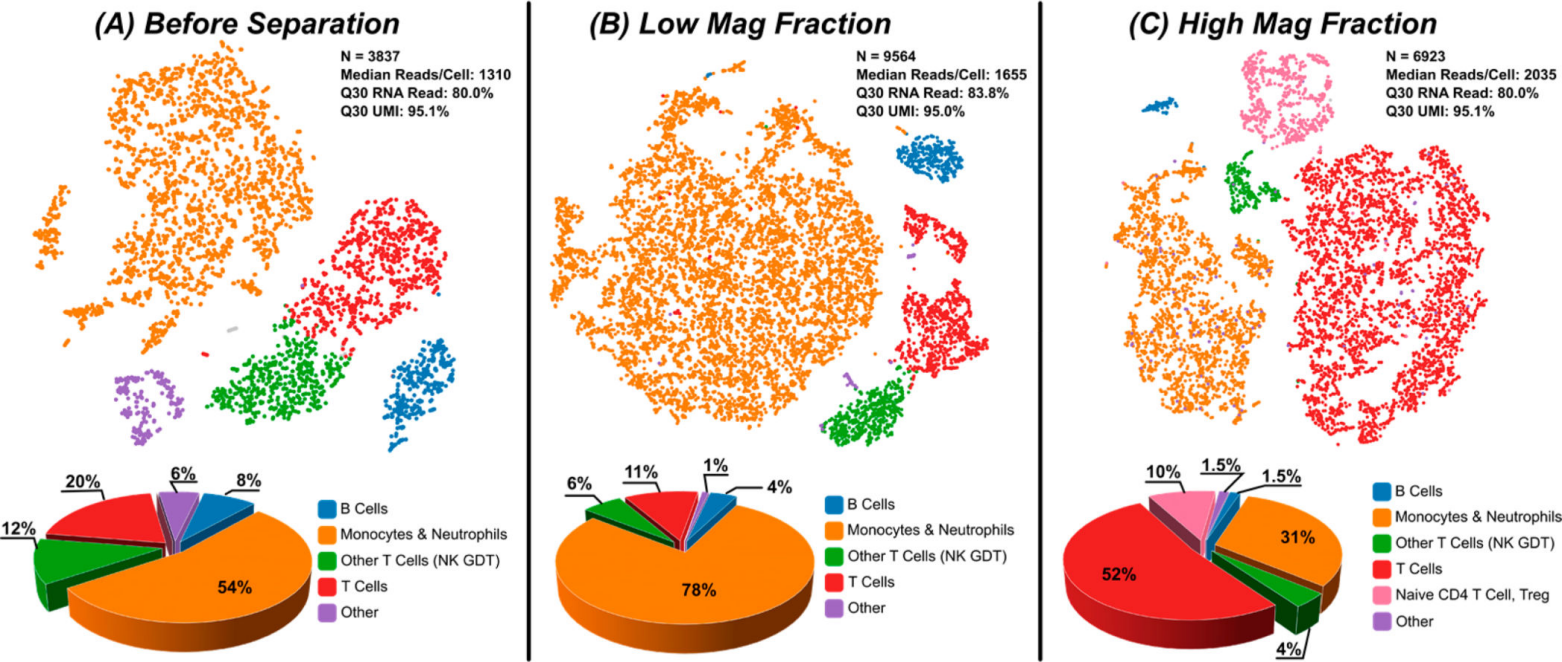
Single-cell RNA seq using a 10× GEM chip was performed with leukocyte samples before separation and after fractionation into LMF and HMF. (**A**) Before separation, leukocytes exhibited expected populations of B cells; monocytes and neutrophils; T cells and their subtypes; and other various cell subpopulations. (**B**) tSNE plot of LMF shows enrichment of monocytes (78%) and some T cells (11%). (**C**) The HMF shows enrichment of T cells (52%) and subpopulations (~14%) with some monocytes and neutrophils (31%). These data confirm that the DMS system is fractionating populations, which have been shown to correlate with CD4 high/low expression and align with published data and scientific publications.

CD4 Hi/Low cells sorted by DMS were processed for single-cell RNA seq to corroborate flow cytometry findings for cell population fractionation and examine gene expression. DMS processing into HMFs and LMFs was performed in the same manner as previously described and then added directly into a 10× Genomics chip. Three conditions were processed through the single-cell RNA seq workflow: (1) unsorted control, (2) LMF, and (3) HMF. Figure 4 shows tSNE plots before and after DMS processing into LMF and HMF populations. Initially the sample consisted of standard cell populations with monocytes and neutrophils at a 54% frequency and total T cells at 32% (CD3+ T cells: 20% and other T cells (natural killer (NK) and γδ T cells: 12%), which was consistent with flow cytometry controls.

After DMS, the LMF contained mostly monocytes and neutrophils (~78%), some T cells and their subpopulations (11%, 6%), and a few B cells (4%). The HMF consisted largely of T cells (52%), T cell subpopulations (4%, 10%), as well as monocytes and neutrophils (31%), and very few B cells (1.5%). This shows that the DMS system is fractionating populations, which correlates with published literature with CD4^Hi^ T cells and CD4^Low^ monocytes [5–7].

**Figure 5.**
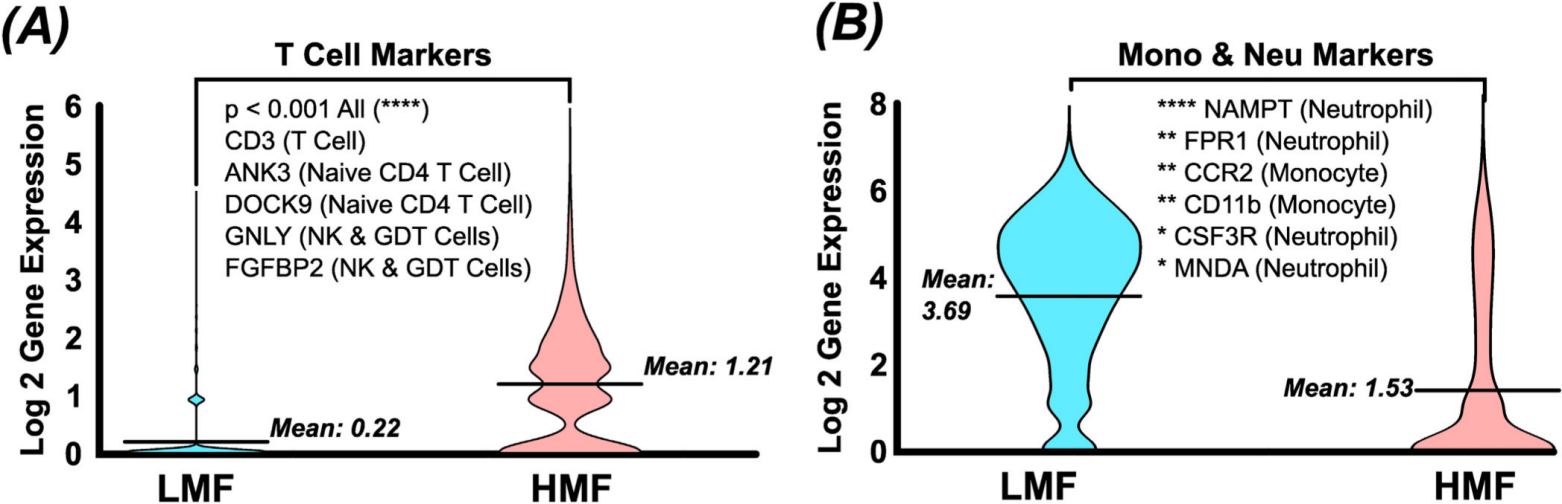
Single-cell RNA sequencing of LMF and DMS fractions. (**A**) Cells in HMF show significantly higher expression of multiple T cell markers compared to the LMF (**B**), which showed significantly higher expression of monocyte and neutrophil markers. These data show that the HMF preferentially enriches T cell populations, while the LMF is preferentially enriching for monocyte and neutrophil populations. Note * *p* < 0.05, ** *p* < 0.01, and **** *p* < 0.0001.

In-depth gene expression analysis of T cell markers and monocyte and neutrophil markers shows a significant difference between the HMF and LMF in terms of population fractionation. Figure 5A shows a comparative violin plot between LMF and HMF, visualizing aggregated gene expression for multiple T cell markers. These markers correlate with T cells and their subpopulations, specifically T cells (CD3), naïve CD4+ T cells (ANK3 and DOCK9), and natural killer and γδ T cells (GNLY and FGFBP2) [22]. As shown, the mean gene expression of T cell markers in the HMF is significantly higher than the LMF (*p* < 0.0001), meaning the HMF is enriching for T cell populations. Conversely, Figure 5B shows that the LMF is enriching monocyte and neutrophil populations all with statistically significant differences (*p* value ranges from <0.05 to <0.0001). These data confirm that the DMS process is successfully fractionating cell types correlating with CD4^Hi^ and CD4^Low^ expression in established literature. These data, in conjunction with the flow cytometry findings, suggest that the DMS system is indeed fractionating for CD4^Hi^ and CD4^Low^ populations and agree with previously published datasets. It is of note that measurement of CD4 surface marker expression with RNA seq data cannot be directly correlated with surface protein levels [23].

### 3.4. Cell-Processing Throughput

**Figure 6.**
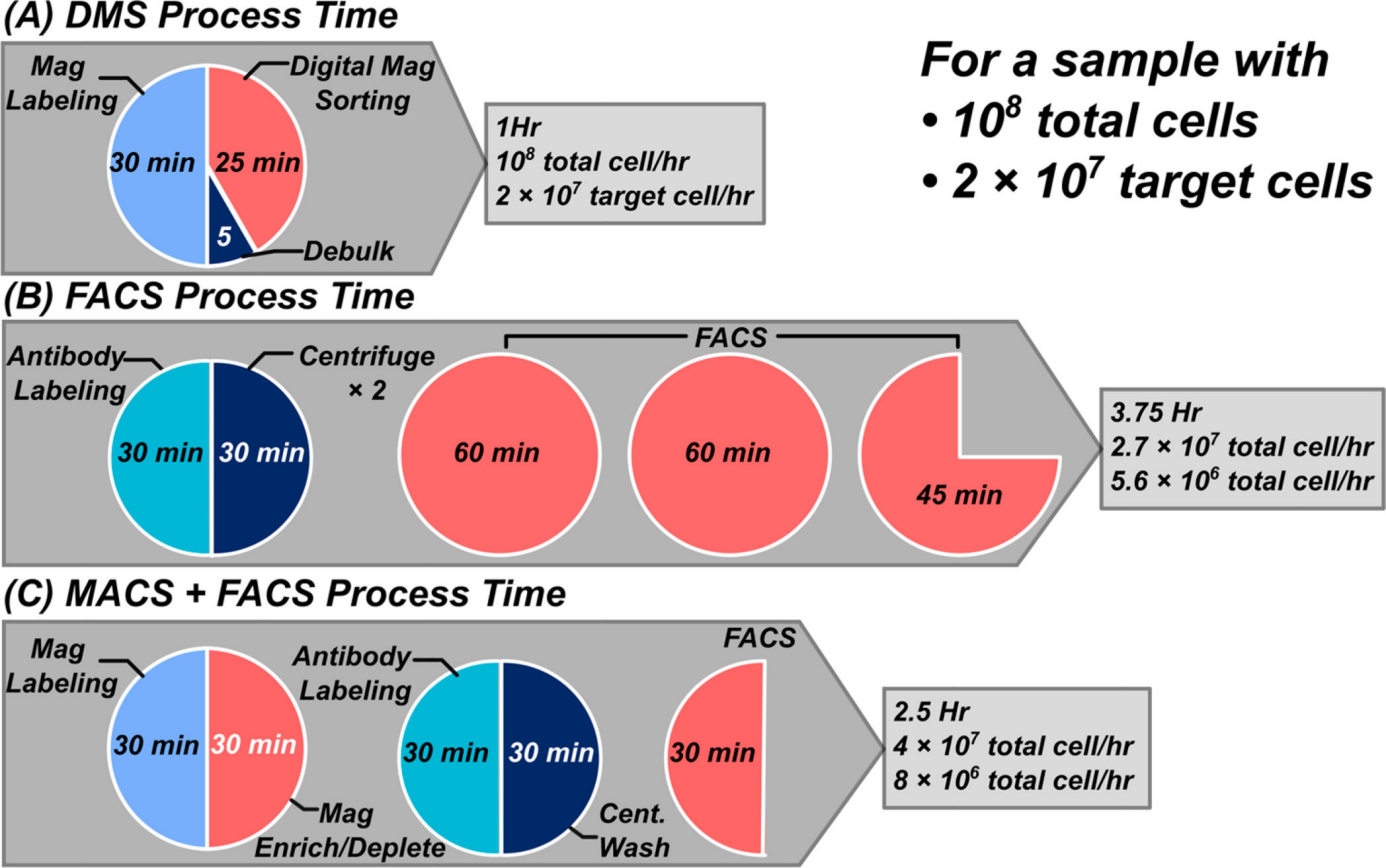
Throughput comparison between DMS, FACS, magnetic enrichment/depletion, and FACS. (**A**) Due to its parallelized nature, DMS can achieve higher throughput processing of antigen density subpopulations. DMS is centrifugation-free and requires 30 min of magnetic labeling, rapid magnetic debulking, or preselection followed by antigen density fractionation via DMS. (**B**) Contrastingly, FACS workflows require all cells in a sample to be analyzed and sorted, which leads to long sort times that negatively impact viability and throughput. A FACS workflow requires antibody labeling and multiple centrifugation and wash steps before proceeding to FACS. Assuming a 1 × 10^4^ cell per second throughput, a sample of 1 × 10^8^ cells would take about 2.7 h to complete. (**C**) Often-times magnetic enrichment or depletion is performed to debulk a sample before FACS. The first magnetic enrichment/depletion is performed by magnetic labeling and pulldown via bulk magnet or column. Then, the sample is processed for FACS isolation. While this FACS + MACS method increases throughput, it requires the addition of multiple sample processing steps, which reduces target cell yield and increases consumable and reagent costs significantly. Typically, a post-sort process evaluation step is performed by sampling cell viability and cell counts with hemocytometers or automated cell counting techniques. Commonly, post-sort cell count and viability checks are performed regardless of sorting platform (DMS, FACS, or MACS + FACS) and typically take ~5 min to complete.

Because of its parallelized nature and streamlined sample processing steps (centrifugation-free), DMS can achieve higher throughput compared to traditional methods for antigen density-based separations such as FACS. Quantification of DMS throughout was characterized by evaluating the total target cells separated over the entire process (magnetic labeling, magnetic preselection/debulking, and DMS). Figure 6 shows a throughput breakdown from sample procurement to completion, assuming a sample of 10^8^ total cells and a target fraction of 2 × 10^7^ target cells. Briefly, a single benchtop DMS cartridge can process 10^8^ total cells containing 2 × 10^7^ target cells and fractionate them into high/low populations between 50 and 60 min (30 min magnetic bead labeling, 5 min magnetic preselection/debulking, and a 20-to-30-minute separation). Assuming a 60 min total process time, this yields a 10^8^ total cell per hour throughput and a 2 × 10^7^ target cell per hour throughput. Compared to flow cytometry (Figure 6B), a sample containing 10^8^ cells would take ~3.75 h (30 min for antibody staining, 2 × 15 min centrifugation and washing steps, and 2.75 h for FACS assuming 10k cells/sec throughput) [24], yielding a 2.7 × 10^7^ total cell per hour throughput.

The main challenge with using FACS to sort large cell quantities is that both target and non-target cells are sorted; in this case, ~80% of sorting time is spent on non-target cells. Often magnetic enrichment or depletion steps are used to debulk non-target cells before running on FACS [25–27]. In this case, a 1 × 10^8^ cell sample with 2 × 10^7^ target cells would take approximately 2.5 h (30 min magnetic labeling and 30 min magnetic pulldown or column sort followed by standard FACS antibody staining and washing 60 min, and FACS 30 min assuming 10k cells/sec throughput on 2 × 10^7^ target cells). This combined magnetic enrichment/depletion and FACS yields a total cell throughput of 4 × 10^7^ total cells per hour throughput, or 8 × 10^8^ target cells per hour, assuming 2 × 10^7^ target cells in the starting fraction. Although a FACS + MACS method enhances throughput, it necessitates multiple additional sample processing steps, which can significantly reduce target cell yield and increase the costs of consumables and reagents. This suggests that the benchtop DMS system can achieve 2× to 3× throughput compared to FACS-based techniques in a single process workflow and can be readily scaled to larger footprints to accommodate larger cell quantities.

## 4. Discussion

In this study, we investigated the potential of digital magnetic sorting (DMS) to fractionate cell populations based on CD4 antigen density. Our results provide strong evidence of the successful fractionation of CD4+ cells into CD4^Hi^ T cells and CD4^Low^ monocytes and neutrophils, confirming the robustness of DMS in accurately sorting cell subsets based on antigen density. Furthermore, the findings were confirmed by multiple assay approaches, specifically flow cytometry and single-cell RNA sequencing, both of which show fractionation of expected CD4^Hi^ T cells and CD4^Low^ monocyte/neutrophil populations and are in agreement with established literature [5–7]. The ability to fractionate cell populations based on antigen density holds at manufacturing scale throughput shows significant promise for improving cell therapy manufacturing. Even at benchtop scale, the DMS platform can process throughputs of 10^8^ total cells per hour, capable of processing 10^8^ total cells from sample to sorted cells in less than 60 min, which is significantly faster than FACS systems (3× faster). DMS’ throughput is comparable to widely utilized magnetic sorting systems such as the Miltenyi^®^ AutoMACS or StemCell Technologies^®^ Robosep. However, the key differentiator for DMS is the capability to fractionate based on antigen density, which is not currently possible with binary magnetic sorters.

**Figure 7.**
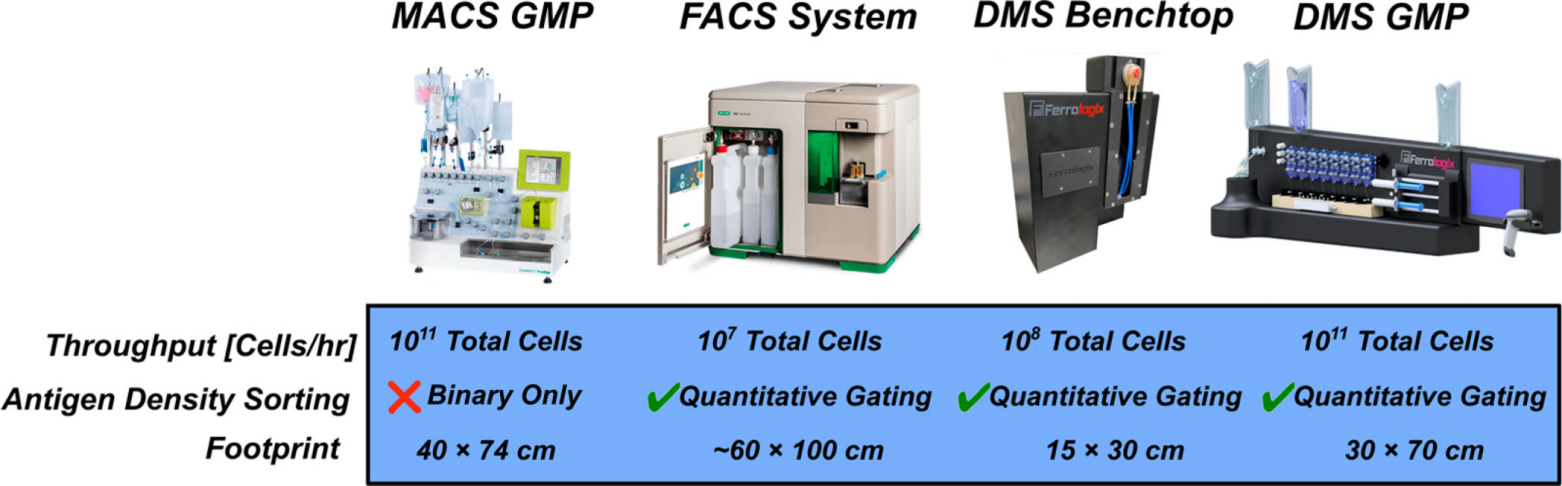
Comparison of DMS and other cell sorting footprints. MACS GMP scale systems for cell therapy production can accommodate large cell quantities but cannot fractionate subpopulations based on antigen density. FACS sorters have quantitative gating capabilities but are too low throughput to accommodate cell therapy production scale. DMS offers a scalable approach to sorting based on antigen density by cartridge parallelization. A single DMS benchtop sorter can achieve a 10^8^ cell/h throughput in a single run. The scaled DMS GMP system has eight parallelized cartridges that operate in semicontinuous batch mode to achieve a total throughput of 10^9^ cells/h.

While a benchtop DMS system is capable of operating at cell therapy process development scales, which generally consist of cell quantities of 10^7^ to 10^8^ total cells [28], cell therapy manufacturing operates at much larger cell quantities of 10^9^ to 10^11^ total cells [29]. Fortunately, the scaling strategy for DMS to operate at cell therapy manufacturing levels is relatively straightforward, where a parallelized cartridge approach can be adopted. Indeed, Ferrologix is actively developing a manufacturing-scale GMP-compliant DMS system (Figure 7) that consists of multiple cartridges processing samples in parallel [30]. The DMS chip has a small footprint (2.5 cm × 6.5 cm), which can be readily scaled to larger footprints for parallel sorting of higher cell quantities. Therefore, a 25 cm × 6.5 cm chip can process 10^9^ total cells in a single run or a whole leukopak (~2 × 10^9^ total cells) in two batch runs. A throughput of ~10^9^ cells/h is comparable to current cell therapy manufacturing magnetic sorting systems but would add the capability to fractionate based on antigen density.

In the larger landscape of cell therapy manufacturing, the cost of production is a major obstacle for wide-scale deployment. Indeed, one of the major cost drivers for cell therapy production is the procurement of cGMP-grade materials [31], of which genetic modification reagents are the costliest (e.g., GMP viral vector and CrispR/CAS9 reagents). One potential solution to reducing costs of cell therapy production is to engineer lower quantities of highly specific cell populations to achieve the same or improved therapeutic response. Recent research in cell therapy production also supports this approach, where lower numbers of higher quality cells are achieving a superior response to traditional heterogenous population approaches and are much lower cost to produce [32,33]. One specific example in support of the lower quantity and higher specialization argument is in the production of natural killer (NK) cell therapies. It has been well established that NK cells split into different phenotypes based on antigen density, where CD56^Hi^ NK cells exhibit higher cytokine activity and cytotoxicity [34] and have higher anti-tumor activity [35]. Isolation and engineering CD56^Hi^ NK cell subpopulation, which represent approximately 10% of the total circulating NK cell fraction, could substantially reduce the total cell quantity in the end therapeutic and therefore the required quantity of reagents needed to produce the cell therapy. Using DMS to fractionate NK cells based on CD56 antigen density can enable the isolation of specific subpopulations with higher therapeutic potential while also reducing the reagent cost to manufacture. CD56^Hi/Low^ fractionation represents one specific example where DMS can potentially improve therapeutic potency and cost reduction with many other potential applications in the production of engineered T cells or stem cell therapeutics.

## 5. Conclusions

In this work, DMS demonstrated successful fractionation of cell populations based on CD4^Hi^ and CD4^Low^ antigen density using differential magnetic binding per cell as a rapid and scalable method for differentiating cell subpopulations. DMS separations characterized by both flow cytometry and single-cell RNA seq show differential CD4 expression between HMFs and LMFs and cell population compositions that are consistent with established literature on CD4^Hi/Low^ leukocyte cell types. The benchtop DMS system also demonstrated higher throughput compared to FACS systems with a clear scaling strategy to accommodate cell quantities associated with cell therapy manufacturing. This capability to fractionate cell types by antigen density in a scalable manner can enable novel precision medicine applications, including specific examples in use today such as NK cell subpopulation enrichment, stem cell subpopulation fractionation, or even enrichment of engineered cells after transduction. Our hope is to utilize this novel technology to reduce the production cost of cell therapies by providing cell therapy developers with a tool to isolate lower numbers of highly specialized cell types that can be engineered more efficiently and with lower reagent consumption.

## 6. Patents

Ferrologix has and continues to develop a patent portfolio of its innovations (please see US20220241797A1).

## Data Availability

Protocols are available on the Cancer Nanotechnology Laboratory (caNanoLab) database.
